# Multidimensional features of sporadic Creutzfeldt-Jakob disease in the elderly: a case report and systematic review

**DOI:** 10.3389/fnagi.2024.1379011

**Published:** 2024-04-09

**Authors:** Jiangfeng Liao, Wenming Hu, Shiheng Chen, Chunyu Huang, Senwei Dong, Wanjin Chen, Xiaochun Chen, Longfei Chen

**Affiliations:** ^1^Department of Neurology and Institute of Neurology of the First Affiliated Hospital, Institute of Neuroscience, and Fujian Key Laboratory of Molecular Neurology, Fujian Medical University, Fuzhou, China; ^2^Department of Neurology, National Regional Medical Center, Binhai Campus of the First Affiliated Hospital, Fujian Medical University, Fuzhou, China; ^3^Department of Neurology, Fuzhou Changle District People’s Hospital, Fuzhou, China; ^4^Department of Neurology, Fujian Medical University Union Hospital, Fuzhou, China

**Keywords:** sporadic Creutzfeldt-Jakob disease, elderly, clinical features, diagnosis biomarkers, neuropathology hallmarks

## Abstract

**Background:**

As a rare neurodegenerative disease, sporadic Creutzfeldt-Jakob disease (sCJD) is poorly understood in the elderly populace. This study aims to enunciate the multidimensional features of sCJD in this group.

**Methods:**

A case of probable sCJD was reported in a 90-year-old Chinese man with initial dizziness. Then, available English literature of the elderly sCJD cases (aged 80 years and over) was reviewed and analyzed. Patients (15 cases) were subdivided and compared geographically.

**Results:**

In the elderly sCJD cohort, the onset age was 84.9 ± 4.5 years and the median disease duration was 6.8 months, with respiratory infection/failure as the commonest death cause. Various clinical symptoms were identified, with cognitive disorder (86.7%) as the commonest typical symptom and speech impairment (66.7%) as the most atypical one. Restricted hyperintensities were reported in 60.0% cases on DWI, periodic sharp wave complexes in 73.3% cases on electroencephalogram, and cerebral hypoperfusion/hypometabolism in 26.7% cases on molecular imaging. The sensitive cerebrospinal fluid biomarkers were total tau (83.3%), 14-3-3 protein (75.0%), and PrP RT-QuIC (75.0%). Neuropathological profiles in the cerebral cortex revealed vacuolar spongiosis, neuronal loss, gliosis, and aging-related markers, with synaptic deposit as the commonest PrP pattern (60.0%). The polymorphic *PRNP* analysis at codon 129 was M/M (90.9%), with MM1 and MM2C as the primary molecular phenotypes. Latency to first clinic visit, hyperintense signals on DWI, and disease duration were significantly different between the patient subgroups.

**Conclusion:**

The characteristics of sCJD are multidimensional in the elderly, deepening our understanding of the disease and facilitating an earlier recognition and better care for this group.

## 1 Introduction

Creutzfeldt-Jakob disease (CJD), a rare but fatal neurodegenerative disorder, is caused by aggregation of misfolded protease-resistant prion protein scrapie (PrP^Sc^) that derives from its normal cellular isoform (PrP^C^) in the brain (Hermann et al., 2021; Liao et al., 2021). Currently, the disease is incurable and death may ensue within months (Staffaroni et al., 2019).

Etiologically, CJD can be divided into three major groups: sporadic, genetic, and acquired (including iatrogenic and variant) types, in which sporadic CJD (sCJD) is the most common (about 90%), with an incidence around 1.5 to 2.0 per million person-year (Hermann et al., 2021). The typical clinical manifestations of sCJD feature rapidly progressive dementia, psychiatric disorders, ataxia, visual symptoms, myoclonus, and akinetic mutism, while the atypical ones include limb weakness, dizziness, speech impairment, and sleep disorder. Its molecular subtypes are defined by both the gene polymorphism of *PRNP* at codon 129 [Methionine (M) or Valine (V)] and the molecular mass of PrP^Sc^ glycoforms [21 kDa (type-1) or 19 kDa (type-2)], which roughly classifies sCJD into six subgroups: MM1, MM2, MV1, MV2, VV1, and VV2 (Parchi et al., 1999). The MM2 type can be further divided into cortical- (MM2C) and thalamic-subtype (MM2T) (Parchi et al., 1999). Patients with MM1 manifest the typical clinical features of sCJD, while those with MM2C often present atypical but characteristic features (Parchi et al., 1999; Iwasaki et al., 2019). Due to the heterogeneity of clinical manifestations and limitations of accessible diagnostic investigations, there is a high risk of misdiagnosing sCJD as a variety of other illnesses.

Although sCJD occurs most commonly in the sixth and seventh decades of life, it also affects much older individuals, up to 6.1% of those aged 80 years and over (Collins et al., 2006; Uttley et al., 2020). Over the past three decades, hundreds of sCJD cases older than 80 years have been observed and analyzed by surveillance systems worldwide (Denouel et al., 2023; Peden et al., 2024). However, no discernible clinical or pathological differences related to the age of patients are identified and limited work has specifically addressed this issue. Therefore, the potential relationship remains ambiguous. Indeed, significant challenges remain for an accurate diagnosis and proper management of these elder individuals, particularly when they present atypical clinical manifestations or are comorbid with other neurodegenerative disorders. Amid this developing trend of an aging society, due attention to the features of sCJD in the elderly is of enormous significance, which may improve our understanding of sCJD and facilitate an early screening in the elderly populace and better management of their late life.

In this study, we reported a case of a 90-year-old Chinese man with sCJD who initially presented dizziness and then conducted a systematic review of all the accessible published sCJD cases aged over 80 years old. Our multidimensional findings may promote our further understandings of sCJD and provide novel insights into the diagnosis and treatment of this disease in the elderly populace.

## 2 Materials and methods

The systematic review was conducted in strict accordance with the Preferred Reporting Items for Systematic Reviews and Meta-Analyses (PRISMA) guidelines standards.^1^ The PRISMA checklist was provided in Supplementary material. The study protocol was registered in PROSPERO (ID: CRD42023483925).

### 2.1 Patient cohort

A 90-years-old Chinese man clinically diagnosed as sCJD was reported in our study. Details of records were collected including medical history, physical examination, magnetic resonance imaging scanning, electroencephalography, and lumbar puncture examinations. Then, we performed literature search with the keywords “sporadic Creutzfeldt-Jakob disease” and “case report” in the PubMed, which retrieved a total of 505 papers (from March 1987 to August 2023). Through manual screening, a total of 15 cases (including 14 from literature and our present case) were enrolled for subsequent analysis. Case selection criteria: (1) reported in English; (2) aged over 80 years old; (3) meeting the World Health Organization (WHO) criteria for probable or definite sCJD (Zerr et al., 2009); (4) without family history of CJD or other forms of dementia. Exclusion criteria: genetic CJD and cases reported in other language or under 80 years of age. The details are summarized in Supplementary Table.

This study was approved by the Ethics Committee of First Affiliated Hospital, Fujian Medical University (MTCA, ECFAH of FMU [2015]084-2). The patient agreed to participate in this study and provided written informed consent.

### 2.2 Imaging analysis

For the reported case, head magnetic resonance imaging (MRI) was performed in our hospital. T1- and T2-weighted, fluid-attenuated inversion recovery (FLAIR), diffusion-weighted imaging (DWI), and apparent diffusion coefficient (ADC) sequences were routinely analyzed. An overall impression of the prion disease was provided by the neuroradiologist. For systemic review, we focused on summaries of the imaging findings on both the DWI and the positron emission tomography (PET) or single-photon emission computed tomography (SPECT).

### 2.3 Electroencephalogram (EEG)

For the reported case, an EEG was performed in our hospital and the report was provided by the neurologist. For systemic review, particular interests focused on the periodic sharp wave complexes (PSWC) (Steinhoff et al., 2004).

### 2.4 Polymerase chain reaction (PCR)

The blood samples were collected and stored at −80°C prior to the analysis. Human genomic DNA was extracted from peripheral blood leukocytes by standard methods. We designed the pre-primer of *PRNP* gene as GCACCCACAGTCAGTGGAAC and the post-primer as GGGCTTGACCAGCATCTCAG. The total PCR reaction system (25 ul) consisted of Thermococcus kodakaraensis (KOD) enzyme (12 ul), ddH2O (10 ul), the pre- and post-primers (1 ul, respectively), and the DNA sample (1 ul). The reaction steps were proceeded at a denaturation temperature of 98°C for 1 min, an annealing temperature of 62°C for 30 s, and an extension temperature of 72°C for 5 min. The latter two steps were repeated 30 times and the temperature at the end was 4°C.

### 2.5 Statistical analysis

The Chi-square test was used to compare differences in rates. The Shapiro–Wilk was used to assess the data distribution normality and the Mann–Whitney *U* test to compare the differences between subgroups. Survival analysis was performed between the male and female patients. Pearson’s correlation test was employed to analyze the correlations. All tests were performed with GraphPad Prism 9.5 software.

## 3 Results

### 3.1 Case report

A 90-year-old Chinese man was admitted to our department, with a chief complaint of dizziness, speech reduction, and memory decline. About six months ago, the patient complained of but paid little attention to repeated dizziness, which gradually aggravated and was accompanied by frequent and urgent urination and increased nocturia. He was admitted to the local clinic and underwent MRI, which reported abnormal signals in the bilateral occipital and left temporal and parietal lobes. The patient was initially diagnosed as acute ischemic stroke and treated with antiplatelet drugs. However, he gradually experienced speech reduction, slow movement, unsteady walking, low mood, loneliness, and longing for companionship. Twenty days before admission, his family noticed that the patient sometimes cried unconsciously, suffered from obvious memory decline, and failed to remember what he ate for meals, recognize his relatives, and perform simple calculations.

The patient had a previous history of hypertension, diabetes, and surgery for bone fractures. His family denied previous fever, epilepsy, head trauma, and any significant family medical history. Neurological examinations showed impairments of calculation, orientation, and short-term memory. Cranial nerve examination was unremarkable. No apparent muscle weakness, parkinsonism, or myoclonus were identified. The Mini-Mental State Examination (MMSE) was 6/30, the Hamilton Depression Scale (HAMD) was 18, and the Hamilton Anxiety Scale (HAMA) was 10.

**FIGURE 1:**
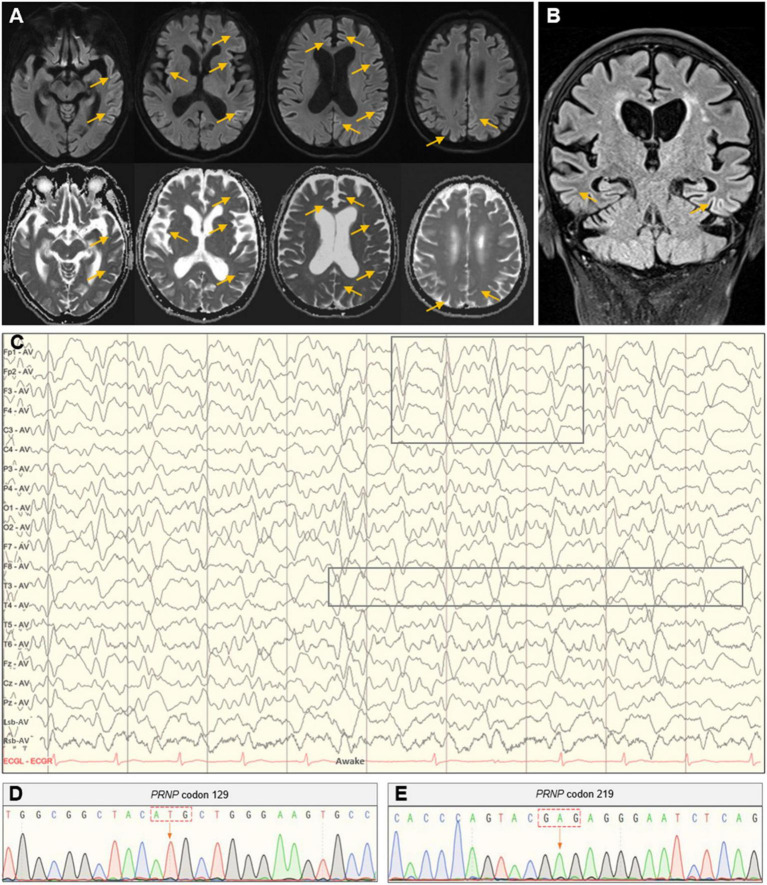
Head MRI, EEG, and *PRNP* gene analysis. **(A)** Axial imaging showed hyperintense signals in the bilateral parietal-occipital and frontal lobes in the DWI with correlating hypointense signals in the ADC map. **(B)** Coronal imaging showed hyperintensities in the bilateral parietal-temporal lobes in the FLAIR sequence. **(C)** The PSWC on EEG. **(D,E)** Polymorphic analysis of the *PRNP* gene at codon 129 and 219.

Routine laboratory examinations of blood, heart, liver, kidney, and thyroid functions reported no remarkable alterations. Other serologic investigations, such as C-reactive protein, electrolyte, ferritin and transferrin, folate, and vitamin B12, were all within normal ranges. The electrocardiogram, chest CT scan, and cardiovascular and abdominal color Doppler ultrasound revealed no explanatory findings either. The magnetic resonance angiography only reported mild arteriosclerosis, and the DWI and FLAIR revealed significant hyperintensities predominantly in the left parietal-occipital and frontal lobes, suggesting prion disease (Figures 1A, B).

Given the clinical manifestations and imaging findings, the possibility of CJD was considered for this patient and further EEG and lumbar puncture were prescribed. The EEG showed synchronous extensibility and high-amplitude (1.5–3.5 Hz) triphasic sharp waves on a background diffused with low-amplitude activities, which was more pronounced in the left hemisphere (Figure 1C). Lumbar puncture found no abnormalities in examinations of biochemistry, flow cytometry, oligoclonal band, immunoglobulin G, and microbial infection in the cerebrospinal fluid (CSF). The 14-3-3 protein in the CSF was negative, and the *PRNP* gene polymorphic analysis showed methionine homozygous (M/M) at codon 129 and glutamate homozygous (E/E) at codon 219 (Figures 1D, E).

This patient was finally diagnosed as probable sCJD. Although he received increased nutrition and cognitive treatments, his general conditions gradually declined and died of severe pneumonia 6.5 months after the onset of symptoms. His family were counseled for a brain biopsy or RT-QuIC to which they declined.

### 3.2 Systematic review of the elderly sCJD patients aged over 80 years old

A total of 15 sCJD patients were included in our systematic review (Table 1). Overall, the proportion of female patients (66.0%) was slightly higher than that of the males. The onset age was 84.9 ± 4.5 years old (within a range of 80–98 years old). Their first clinic visit was 4.7 ± 4.4 weeks (within a range of 0.3–16 weeks) after the onset of symptoms. About 60.0% (9/15) of the cases had definite past medical histories, most reporting hypertension and diabetes. Of note, 40.0% of the cases died of respiratory infection or failure, suggesting respiratory impairment as the most possible death cause for the elderly sCJD population.

**TABLE 1:** Clinical features of the elderly sCJD patients aged over 80 years old.

Clinical features	Total	Southeast Asia	Western Europe	*p*-value
Number of cases	15	8	7	
Sex (female)	9/15 (66.0%)	3/8 (37.5%)	6/7 (85.7%)	0.0572
Onset age (y)	84.9 ± 4.5	84.6 ± 2.9	85.1 ± 6.1	0.7166
Time to first clinic visit (w)	4.7 ± 4.4	6.1 ± 4.6	3.2 ± 3.9	0.0181
**Clinical symptoms**				
Onset symptoms (atypical)	7/15 (46.7%)	4/8 (50.0%)	3/7 (42.9%)	0.7821
Cognitive impairment	13/15 (86.7%)	7/8 (87.5%)	6/7 (85.7%)	0.9192
Akinetic mutism	7/15 (46.7%)	4/8 (50.0%)	3/7 (42.9%)	0.7821
Myoclonus	10/15 (66.7%)	5/8 (62.5%)	5/7 (71.4%)	0.7144
Extrapyramidal symptoms	7/15 (46.7%)	2/8 (25.0%)	5/7 (71.4%)	0.0721
Ataxia	8/15 (53.3%)	3/8 (37.5%)	5/7 (71.4%)	0.1888
Visual impairment	5/15 (33.3%)	2/8 (25.0%)	3/7 (42.9%)	0.4642
Seizure	4/15 (26.7%)	3/8 (37.5%)	1/7 (14.3%)	0.3104
Pyramidal signs	6/15 (40.0%)	4/8 (50.0%)	2/7 (28.6%)	0.3980
**Psychiatric symptoms**				
Depressive mood	4/15 (26.7%)	3/8 (37.5%)	1/7 (14.3%)	0.3104
Hallucination	3/15 (20.0%)	2/8 (25.0%)	1/7 (14.3%)	0.6048
Limb weakness/numbness	5/15 (33.3%)	4/8 (50.0%)	1/7 (14.3%)	0.1432
Speech impairment	10/15 (66.7%)	5/8 (62.5%)	5/7 (71.4%)	0.7144
Sleep disorder	4/15 (26.7%)	2/8 (25.0%)	2/7 (28.6%)	0.8760
Dizziness/headache	3/15 (20.0%)	2/8 (25.0%)	1/7 (14.3%)	0.6048
Urinary incontinence	2/15 (13.3%)	1/8 (12.5%)	1/7 (14.3%)	0.9192
**Auxiliary examination**				
MRI imaging (DWI)	9/15 (60.0%)	7/8 (87.5%)	2/7 (28.6%)	0.0201
PET/SPECT imaging	4/15 (26.7%)	2/8 (25.0%)	2/7 (28.6%)	0.8760
EEG (PSWC)	11/15 (73.3%)	5/8 (62.5%)	6/7 (85.7%)	0.3104
**CSF examination**				
14-3-3 protein	9/12 (75.0%)	4/6 (66.7%)	5/6 (83.3%)	0.5050
PrP RT-QuIC	3/4 (75.0%)	2/3 (66.7%)	1/1 (100%)	0.5050
Total tau protein	5/6 (83.3%)	3/4 (75.0%)	2/2 (100%)	0.4386
Brain autopsy	10/15 (66.7%)	4/8 (55.6%)	6/7 (87.5%)	0.1432
*PRNP* analysis (M/M at codon 129)	10/11 (90.9%)	6/6 (100%)	4/5 (80.0%)	0.2521
Disease duration (m)^#^	6.8 ± 10.6	11.0 ± 13.5	2.1 ± 1.1	0.0073
**Death cause**				
Respiratory infection/failure	6/15 (40.0%)	4/8 (50.0%)	2/7 (28.6%)	0.3980
Cardiac rupture	1/15 (6.7%)	1/8 (12.5%)	0/7 (0%)	0.3329

y, year; w, week; m, month; ^#^, after the onset of symptoms; DWI, diffusion-weighted magnetic resonance imaging; PET, positron emission tomography; SPECT, single-photon emission computed tomography; EEG, electroencephalography; PSWC, periodic sharp wave complexes; CSF, cerebrospinal fluid; PrP, prion protein; RT-QuIC, Real-time Quaking Induced Conversion; *PRNP*, the gene encoding for PrP; M, methionine. Data are presented as rate or mean ± SD.

During the disease course, typical manifestations of sCJD were cognitive impairment (mainly memory decline) (13/15, 86.7%), myoclonus (10/15, 66.7%), ataxia (8/15, 53.3%), extrapyramidal (mainly bradykinesia and rigidity) or akinetic mutism (7/15, 46.7%, for both), pyramidal signs (6/15, 40.0%), visual impairment (5/15, 33.3%), seizures or depression (4/15, 26.7%), and hallucination (3/15, 20.0%). Meanwhile, the initial onsets with atypical symptoms accounted for 46.7% (7/15) of the patients. Atypical symptoms were also commonly identified in the elderly cohort, including speech impairment (mainly dysarthria) (10/15, 66.7%), limb weakness or numbness (5/15, 33.3%), sleep disorder (4/15, 26.7%), dizziness or headache (3/15, 20.0%), and urinary incontinence (2/15, 13.3%).

Of the 15 sCJD patients, only 3 (20.0%) of them (including our case) underwent the MMSE assessment. In terms of auxiliary examinations, blood tests and head CT scanning did not reveal specific changes. The DWI detected hyperintensities in the cerebral cortex or basal ganglia in 60.0% (9/15) of the patients, with unilateral hyperintensity in the cerebral cortex as the most common pattern. Molecular imaging tests, including PET-CT and SPECT, were employed in four cases, which revealed hypoperfusion or hypometabolism in certain brain regions, including cerebral cortex, thalamus, and cerebellum (Table 2). EEG examination was performed for all patients, which reported the presence of PSWC in 73.3% (11/15) of them on the basis of synchronous or lateralized periodic discharges. Lumber puncture was conducted in 12 patients for examinations of the CSF, which found increased total tau proteins in 5 cases (83.3%), positive detection of 14-3-3 protein in 9 cases (75.0%), and PrP protein [using Real-time Quaking Induced Conversion (RT-QuIC)] in 3 cases (75%). An increased expression of neuron specific enolase (NSE) in the CSF was only reported in 2 patients from Southeast Asia.

**TABLE 2:** Molecular imaging findings of the elderly sCJD patients aged over 80 years old.

Age (y)	Sex	Inspection method	Results	References
83	M	^99m^Tc-ECD-SPECT	Decreased regional cerebral blood flow in the bilateral frontal, parietal, and right posterior cortices	Hayashi et al., 2019
87	M	SPECT	Severe hypoperfusion in the frontal, temporal, and parietal lobes and the thalamus of the left hemisphere	Hirao et al., 2017
80	M	1. ^18^F-FDG-PET; 2. ^18^F-florbetaben PET	1. A left frontal-parietal, bilateral thalamus and cerebellar hypometabolism; 2. Generally higher tracer uptake in white matter than in gray matter, with similar in white and gray matter of the left parietal and right superior temporal lobes	Matias-Guiu et al., 2017
81	F	^18^F-FDG-PET	Significant hypometabolism in the left occipital lobe and adjacent left parietotemporal and precuneal cortex	Obergassel et al., 2020

y, years old; M, male; F, female; ECD, ethyl cysteinate dimer; FDG, fluorodeoxyglucose; SPECT, single-photon emission computed tomography; PET, positron emission tomography.

**FIGURE 2:**
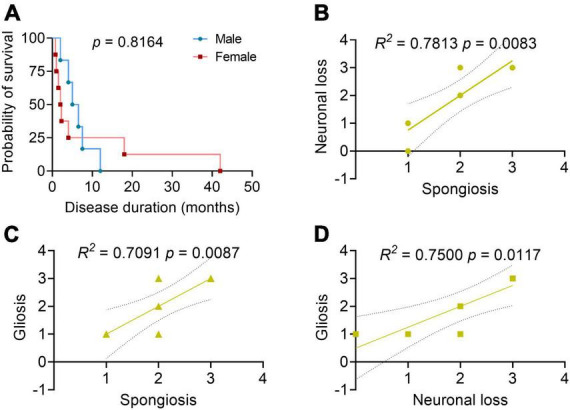
Patient survival analysis and correlation analyses between the neuropathological profiles. **(A)** Survival analysis between the male and female patients with sCJD. *N* = 6 for male and *N* = 8 for female. Log-rank test. **(B–D)** Correlation analyses between the neuropathological profiles. 0, negative; 1, mild; 2, moderate; 3, severe; 4, very severe; the solid line: regression; the dashed lines: 95% confidence. Pearson’s correlation test.

The disease duration of sCJD in the elderly patients was 6.8 ± 10.6 months (within a range of 0.8–42 months). Among them, 40.0% (6/15) died of respiratory infection or failure, 6.7% (1/15) of cardiac rupture, and the others of unknown causes. There were no significant differences found in the survival duration between the male and female sCJD patients (Figure 2A). Brain autopsy followed by neuropathological examinations was performed in 10 patients (Table 3). Six patients macroscopically showed varied cerebral atrophy, and microscopic examinations were performed across different brain regions, such as cerebral cortex, basal ganglia, cerebellum, and brainstem. Among them, the cerebral cortex was the most affected area, in which almost all cases reported, to varying degrees, vacuolar spongiform changes, neuronal loss, and gliosis. Significant correlations were found between the three pathological features [spongiosis and neuronal loss (*R*^2^ = 0.7813, *p* = 0.0083); spongiosis and gliosis (*R*^2^ = 0.7091, *p* = 0.0087); neuronal loss and gliosis (*R*^2^ = 0.7500, *p* = 0.0117)] (Figures 2B–D). Histochemical staining revealed single PrP deposit or different types of mixed PrP deposits, according to their presenting frequency from high to low in this order, including synaptic (60.0%), perivacuolar (40.0%), plaque-like (20.0%), and coarse and granular (10.0%, for both) types. Evidence of tau pathology was also reported in 8 cases and that of beta amyloid pathology in 4 cases, while no α-synuclein pathology was reported.

**TABLE 3:** Neuropathological profiles in the cerebral cortex of the elderly sCJD patients aged over 80 years old.

	Microscopic findings							
Cerebral atrophy	Spongiosis	Neuronal loss	Gliosis	PrP	Tau	Aβ	α-Syn	References
++	++ (small-intermediate vacuoles)	+++	+++	Pl, G, S	P	P	N	Uchino et al., 2023
+	+ (fine-large confluent vacuoles)	–	+	Pv, S	P	P	N	Iwasaki et al., 2019
+++	+++	+++	+++	NA	P	N	NA	Kawashima et al., 1999
+	+ (large confluent vacuoles)	+	+	Pv, C	P	N	N	Hayashi et al., 2019
NA	NA	NA	NA	NA	P	NA	NA	Gonzalez-Martinez et al., 2021
NA	+++	NA	+++	S	P	P	NA	Buganza et al., 2009
+	+++	+++	+++	Pl, Pv, S	P	P	NA	Paquet et al., 2008
NA	+	NA	NA	S	NA	NA	NA	Beaudry et al., 2002
+	++ (confluent vacuoles)	++	++	Pv	NA	NA	NA	de Silva et al., 1997
NA	++ (small-medium vacuoles)	++	+	S	P	N	N	Matias-Guiu et al., 2017

–, none; +, mild; ++, moderate; +++, severe (diffuse); NA, not available; P, positive detection; N, negative detection; Pl, plaque-like; Pv, perivacuolar; G, granular; S, synaptic; C, coarse (rough plaque).

In the present cohort, polymorphic analysis of the *PRNP* gene at codon 129 was conducted in 11 patients, 10 with M/M (90.9%) and only 1 with V/V (9.1%). Further Western blotting analysis demonstrated the presence of both the PrP^Sc^ types (including type 1, type 2, and their hybrids) and the variably protease-sensitive PrP (VPSPr). Taken together, these elderly sCJD cases displayed relatively diverse molecular subtype profiles, encompassing MM1 (2/11), MM2C, MM1+2C, VV2, and MM-VPSPr.

Given that patients from different regions may display distinct clinical features, the current cohort was divided into the Southeast Asia subgroup and Western Europe subgroup. We compared the data and analyzed their differences. Specifically, no remarkable significances were found in terms of the sex ratio, onset age, clinical manifestations, and varied neuropathological profiles. Patients from Southeast Asia had a disease duration almost five times longer than those from Western Europe (*p* = 0.0073) despite their late first clinic visit (*p* = 0.0181). Hyperintensities on DWI were more frequent in patients from Southeast Asia than those from Western Europe (*p* = 0.0201).

## 4 Discussion

In this study, we reported a case of probable sCJD in a 90-year-old Chinese man initially presenting dizziness. Such diagnosis was made as he had rapidly progressive dementia, ataxia, obvious diagnostic DWI hyperintensity, and typical EEG findings. The present case indicates that the elderly sCJD patients may be misdiagnosed when initially onset with atypical symptoms and that timely head DWI and EEG can facilitate its early identification. Moreover, we performed a systematic review, including 15 cases aged over 80 years old, to delineate their clinical, imaging, neuropathological, and molecular characteristics. This study not only suggests avenues for future research to deepen our understanding of CJD, but sheds new lights on more rational treating or managing strategies for the elderly sCJD.

In the present cohort, the average onset age was 84.9 years old, which is significantly advanced when compared with a mean age of 67 years in a total sCJD cohort (Uttley et al., 2020); the proportion of female patients was about 66.0%, similar to those reported in other cohorts (Collins et al., 2006; Staffaroni et al., 2019). They had an average disease duration of 6.8 months (after the symptom onset), higher than 3 months reported in another elderly cohort (de Silva et al., 1997) and 5 months in a total sCJD study (Collins et al., 2006). We noted that respiratory infection/failure is the most likely death cause for the elderly sCJD, highlighting the importance of early respiratory management for this population. Although a multivariate analysis indicates that an increment of 10 years in the onset age may increase the risk of death by about 30% (Tam et al., 2023), the underlying relationship still awaits further investigations. Intriguingly, when our cohort was divided into two subgroups, hyperintensities on DWI were more frequent in patients from Southeast Asia than those from Western Europe. The disease duration in the Southeast Asian patients (11.0 months) was almost five times longer than that in the Western European ones (2.1 months), although the former delayed their first physician consultation more. These findings to certain extent reflect the geographic differences of the elderly sCJD.

The onset symptoms of the current elderly sCJD cohort are similar to those described in total sCJD studies (Zerr et al., 2009; Hermann et al., 2021). Specifically, the frequency of typical symptoms during the disease course in the present cohort from high to low are cognitive impairment (86.7%), myoclonus (66.7%), ataxia (53.3%), extrapyramidal and akinetic mutism (46.7%), pyramidal signs (40.0%), visual impairment (33.3%), and seizures (26.7%). Unlike the psychiatric symptom spectrums reported in another elderly sCJD group, which mainly presents personality and behavioral changes (de Silva et al., 1997), we here emphasize the presence of depression (26.7%) and hallucination (visual or auditory) (20.0%). Notably, initial onsets involving atypical symptoms are of particular interest and found in 46.7% of our patients, seemingly higher than that in another elderly sCJD group (de Silva et al., 1997). During the disease course, the frequency of atypical symptoms from high to low in the present cohort are speech impairment (66.7%), limb weakness or numbness (33.3%), sleep disorder (26.7%), dizziness or headache (20.0%), and urinary incontinence (13.3%). While no significant differences in the clinical manifestations were evident between patients from Southeast Asia and those from Western Europe.

Brain imaging is essential for the diagnosis of sCJD and its differential diagnosis with other neurological diseases, such as ischemia, encephalitis, and neoplasia (Hermann et al., 2021). The typical MRI pattern of CJD occurs in very early disease stages, showing restricted diffusion in at least two cortical regions (“ribboning”) or in the caudate nucleus, followed by putamen and thalamus (Hermann et al., 2021). The present study revealed that 60.0% of the elderly sCJD patients showed hyperintensity in the cortex or basal ganglia on DWI, which is significantly lower than that of previous studies (Bizzi et al., 2020; Hermann et al., 2021). Functionally, consistent with previous studies extensively indicating the correlations between FDG-PET and the clinical symptoms of sCJD (Renard et al., 2017), the present study also found hypoperfusion or hypometabolism detected by PET or SPECT in several brain regions, including cerebral cortex, thalamus, and cerebellum.

Periodic sharp wave complexes (PSWC) on EEG has historically been considered as a hallmark finding in patients with sCJD. Morphologically, the typical PSWC refers to simple sharp waves or complexes with mixed spikes, polyspikes, and slower waves that last for 100–600 ms, recurring every 0.5–2 s in a background of generalized low voltage slowing (Wieser et al., 2006). The present study reported PSWC in 73.3% of the elderly sCJD patients during the disease course, which is higher than 58.4% in the total sCJD (Collins et al., 2006). Given that PSWC often presents at the late stages of sCJD and may be negative in the early stages, an ambulatory EEG monitoring may be encouraged during the whole disease course. Although such periodic pattern has been partially explained by the dendritic membrane fusion of the affected neurons (Wieser et al., 2006), the specific mechanisms still await further investigations.

Current studies of fluid biomarkers of sCJD are in full swing (Staffaroni et al., 2019; Hermann et al., 2021), but only 14-3-3 protein and RT-QuIC are enrolled in diagnostic criteria. However, the present study found that the most sensitive biomarkers in the CSF of the elderly sCJD was total tau (83.3%), highlighting the critical role of tau protein for its early identifications. Indeed, several sCJD-related CSF surrogate biomarkers, such as neurofilament light chain, S100β, and NSE, have also been identified over the past two decades. We also evidenced the upregulation of NSE in the CSF of two elderly sCJD patients from Southeast Asia. Considering the diverse clinical and molecular phenotypes of sCJD, developing a scoring system with multiple CSF biomarkers would be a feasible approach for disease diagnosis and surveillance. Meanwhile, further investigations into blood biomarkers for sCJD may provide more convenient but less invasive alternatives for early examination and recognition.

Although a definite diagnosis of sCJD requires neuropathological confirmations, the autopsy rate has declined in recent years in line with the increased sensitivity of current in-life diagnostic criteria (Tam et al., 2023). Due to the transmissible nature of sCJD, brain biopsy may not be a recommended routine for the mere purpose of diagnosis and little literature focuses on its neuropathological characteristics. The present study found that 66.7% of the elderly sCJD patients showed complete or partial neuropathological results, featuring, to varying degrees, spongiosis, neuronal loss, and gliosis mostly in the cerebral cortex. Further immunohistochemical studies indicate the synaptic-type as the commonest (60.0%) pattern of PrP deposits in the cerebral cortex where several aging-related markers, such as tau and beta amyloid, are co-existed. Our previous work has indicated that the increased levels of peroxiredoxin 6 may be an alternative pathological hallmark for sCJD (Liao et al., 2021). Although the appearance of tau pathology may be attributed to the neuronal dysfunction in sCJD itself, the influence of comorbidity with other neurodegenerative or cerebrovascular diseases cannot be completely excluded. Anyway, this study may provide vital references for the neuropathological studies of sCJD involving different age stages.

The polymorphic analysis of *PRNP* gene plays an important role in the molecular subtyping of sCJD (Parchi et al., 1999). In the present cohort, a total of 11 patients received *PRNP* gene analysis at codon 129, with M/M accounting for 90.9% (10/11) of the patients, which is significantly higher than 66.1% reported in a previous total sCJD study (Collins et al., 2006). Together with the Western blotting analysis for identification of the PrP^Sc^ types, the MM1 and MM2C may be as the most common subtypes for the elderly sCJD. It would be of particular interesting to dissect the relationships between the molecular subtypes and their clinicopathological phenotypes.

Still, there are certain limitations awaiting further improvements. Although the diagnosis of our reported case appears highly probable sCJD, the document lacks identifications of various point or insert mutations of the *PRNP* gene, like E200K and V201I, which are frequently found in patients without a familial history. For the systematic review: (1) the limited small number of enrolled patients may impede the generalizability of our findings; (2) our analysis may be subject to a potential ascertainment bias due to the utilization of published case reports, whose publication is likely published due to their atypical clinical or pathological manifestations; (3) some potential differences in the clinical manifestations of sCJD may surface at different disease stages (early/late) and should be compared. However, despite the absence of a parallel comparison with younger cohorts of sCJD patients, we have attempted to compare our data with previous results reported in a young sCJD cohort (Boesenberg et al., 2005) in the discussion section.

## 5 Conclusion

This study reports a case of a 90-year-old Chinese man who presented initial onset with dizziness, which was finally diagnosed as probable sCJD. This case highlights the necessity of differentiating sCJD in patients with atypical symptoms and the importance of timely head DWI and EEG for its early recognition. Our systematic review further summarizes multidimensional features of sCJD in the elderly, which not only suggests avenues for future research to deepen our understanding of sCJD, but facilitates its early recognition in this populace and better management for their late life.

## Data availability statement

The original contributions presented in this study are included in the article/Supplementary material, further inquiries can be directed to the corresponding authors.

## Ethics statement

The studies involving humans were approved by the Ethics Committee of First Affiliated Hospital, Fujian Medical University. The studies were conducted in accordance with the local legislation and institutional requirements. The human samples used in this study were acquired from the peripheral blood of patients. Written informed consent for participation was not required from the participants or the participants’ legal guardians/next of kin in accordance with the national legislation and institutional requirements. Written informed consent was obtained from the individual(s) for the publication of any potentially identifiable images or data included in this article.

## Author contributions

JL: Conceptualization, Data curation, Formal analysis, Funding acquisition, Investigation, Methodology, Project administration, Writing – original draft, Writing – review & editing. WH: Formal analysis, Investigation, Methodology, Writing – review & editing. SC: Investigation, Methodology, Writing – review & editing. CH: Investigation, Methodology, Validation, Writing – review & editing. SD: Data curation, Methodology, Resources, Validation, Writing – review & editing. WC: Project administration, Supervision, Writing – review and editing. XC: Project administration, Supervision, Writing – review & editing. LC: Conceptualization, Project administration, Resources, Writing – review & editing.

## Footnotes

1
http://www.prisma-statement.org/
