# Corrigendum: Multidimensional features of sporadic Creutzfeldt-Jakob disease in the elderly: a case report and systematic review

**DOI:** 10.3389/fnagi.2025.1565464

**Published:** 2025-02-07

**Authors:** Jiangfeng Liao, Wenming Hu, Shiheng Chen, Chunyu Huang, Senwei Dong, Wanjin Chen, Xiaochun Chen, Longfei Chen

**Affiliations:** ^1^Department of Neurology and Institute of Neurology of the First Affiliated Hospital, Institute of Neuroscience, and Fujian Key Laboratory of Molecular Neurology, Fujian Medical University, Fuzhou, China; ^2^Department of Neurology, National Regional Medical Center, Binhai Campus of the First Affiliated Hospital, Fujian Medical University, Fuzhou, China; ^3^Department of Neurology, Fuzhou Changle District People's Hospital, Fuzhou, China; ^4^Department of Neurology, Fujian Medical University Union Hospital, Fuzhou, China

**Keywords:** sporadic Creutzfeldt-Jakob disease, elderly, clinical features, diagnosis biomarkers, neuropathology hallmarks

In the published article, there was an error in the **Funding** statement. The funding number for the National Natural Science Foundation of China was incorrectly displayed as “8187106”. The correct funding number is “82301707.” The corrected Funding statement appears below.

